# Mutations in DNA repair genes are associated with increased neoantigen burden and a distinct immunophenotype in lung squamous cell carcinoma

**DOI:** 10.1038/s41598-019-39594-4

**Published:** 2019-03-01

**Authors:** Young Kwang Chae, Jonathan F. Anker, Michael S. Oh, Preeti Bais, Sandeep Namburi, Sarita Agte, Francis J. Giles, Jeffrey H. Chuang

**Affiliations:** 1 Northwestern University Feinberg School of Medicine, Chicago, IL 60611 USA; 2 Robert H. Lurie Comprehensive Cancer Center of Northwestern University, Chicago, IL 60611 USA; 3 The Jackson Laboratory for Genomic Medicine, Farmington, CT 06030 USA; 4 Department of Genetics and Genome Sciences, University of Connecticut Health, Farmington, CT 06032 USA

## Abstract

Deficiencies in DNA repair pathways, including mismatch repair (MMR), have been linked to higher tumor mutation burden and improved response to immune checkpoint inhibitors. However, the significance of MMR mutations in lung cancer has not been well characterized, and the relevance of other processes, including homologous recombination (HR) and polymerase epsilon (POLE) activity, remains unclear. Here, we analyzed a dataset of lung squamous cell carcinoma samples from The Cancer Genome Atlas. Variants in DNA repair genes were associated with increased tumor mutation and neoantigen burden, which in turn were linked with greater tumor infiltration by activated T cells. The subset of tumors with DNA repair gene variants but without T cell infiltration exhibited upregulation of TGF-β and Wnt pathway genes, and a combined score incorporating these genes and DNA repair status accurately predicted immune cell infiltration. Finally, high neoantigen burden was positively associated with genes related to cytolytic activity and immune checkpoints. These findings provide evidence that DNA repair pathway defects and immunomodulatory genes together lead to specific immunophenotypes in lung squamous cell carcinoma and could potentially serve as biomarkers for immunotherapy.

## Introduction

Immune checkpoint inhibitors have reshaped the landscape of treatment for multiple cancers, including squamous cell carcinoma (SCC) of the lung and other types of non-small cell lung cancer (NSCLC)^1,2^. These treatments inhibit immune regulatory molecules such as cytotoxic T-lymphocyte-associated protein 4 (CTLA-4), programmed death 1 (PD-1), and programmed death ligand 1 (PD-L1), which normally function to suppress immune cell activity^3,4^. Blocking immune checkpoints with therapeutic antibodies can augment the anti-tumor immune response, thereby providing the mechanistic basis for immunotherapy. In NSCLC, treatment with immune checkpoint inhibitors has yielded dramatic results with improved clinical response in comparison to standard chemotherapy in certain subpopulations of patients^5,6^.

The specificity of the immune response promoted by these therapies is dependent on neoantigens, which are immunogenic cancer-related peptides formed by distinct somatic mutations in tumor cells^7^. The unique epitopes of these neoantigens are able to elicit a tumor-specific immune response^8^, which can then be amplified by the immune-activating actions of immunotherapy. Neoantigens have been associated with improved clinical response to inhibitors of CTLA-4^9,10^, PD-1^11^, and PD-L1^12^. In many solid tumors, deleterious mutations in DNA repair genes can drive a substantial increase in the number of neoantigens^13^. Deficient DNA repair has accordingly been associated with improved clinical responses to PD-1 blockade. Specifically, insufficiencies in mismatch repair (MMR) conferred greater clinical benefit with pembrolizumab in patients with colorectal cancer^14^, as well as in a study of multiple solid tumor types^15^. These results have now led to the landmark FDA approval for PD-1 inhibitors in MMR-deficient tumors, which represents a paradigm-altering shift towards oncologic treatments centered on molecular profile^15^.

Several other DNA repair pathways have been implicated in contributing to neoantigen load. In an analysis of NSCLC patients, mutations in *POLD1, POLE*, and *MSH2* were identified in tumors with the highest neoantigen burden^11^, which in turn correlated with improved response to PD-1 inhibitors. Further, endometrial cancers with polymerase epsilon (*POLE*) mutations contained increased neoantigen burden and PD-L1 expression^16^, and cases of exceptional responders to immunotherapy have been reported with these mutations^17^. Similarly, alterations in the homologous recombination (HR) apparatus, such as *BRCA1* and *BRCA2* mutations, were associated with higher neoantigen load and increased overall survival after anti-PD-1 treatment^18^.

Though DNA repair mutations have been shown to be relevant to immunotherapy response in a variety of solid tumors, limited data exists detailing the importance of these pathways in lung cancer. We had previously utilized datasets from The Cancer Genome Atlas (TCGA) to demonstrate that DNA repair status was strongly associated with tumor neoantigen burden and immune cell infiltration in lung adenocarcinoma^19^. We hypothesized that a comparable relationship would be elucidated in squamous cell carcinoma (SqCC) of the lung, and that mutations in DNA repair pathways could thus function as biomarkers predictive of response to immune checkpoint blockade.

## Results

### Tumors with DNA repair pathway mutations have increased mutational and neoantigen burden

**Figure 1:**
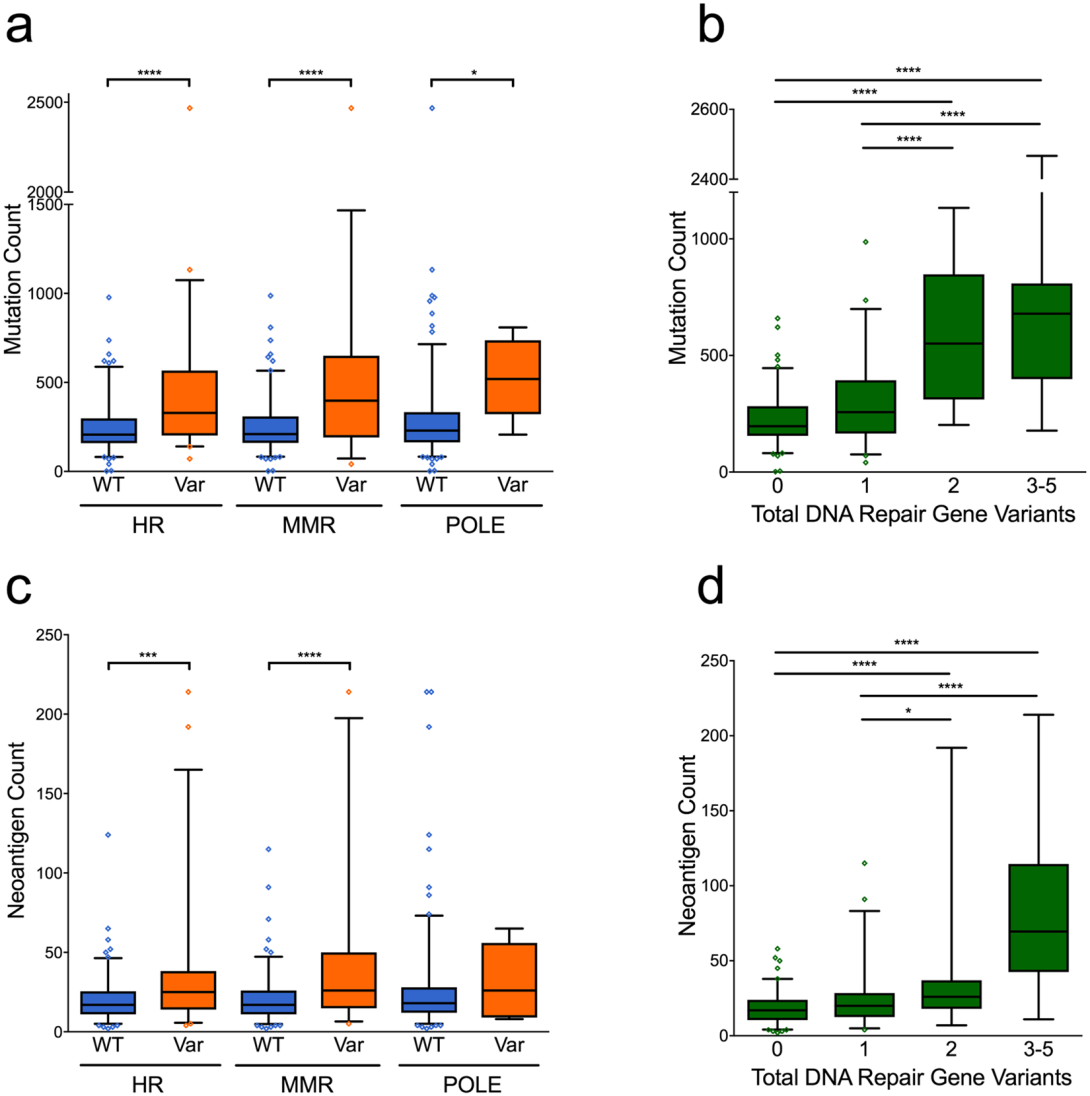
DNA repair gene variants are associated with increased mutation and neoantigen count. (**a**) Presence of somatic variants in homologous recombination (HR), mismatch repair (MMR) or polymerase epsilon (POLE) were associated with increased mutation burden. (**b**) Mutation count increases with higher number of DNA repair gene variants. (**c**) Neoantigen burden similarly was associated with DNA repair gene variants and (**d**) with the number of affected genes. Statistical analysis completed with Student’s t-test (**a**,**c**) and one-way ANOVA with Tukey’s test for multiple comparisons (**b**,**d**), *p < 0.05, **p < 0.01, ***p < 0.001, ****p < 0.0001.

To study the effect of DNA repair gene mutations on tumor mutation burden (TMB) in lung SqCC, we analyzed 178 annotated samples from TCGA^20^. We evaluated tumors for somatic variants in genes related to MMR, HR, or in *POLE*, and identified changes predicted to be deleterious by the SIFT^21^ and CADD v1.4^22^ scoring systems. Tumors with defects in MMR and HR had a significantly higher number of overall mutations (Student’s t-test, p < 0.0001 for both; Fig. 1a). Within HR genes, *BRCA1* and *BRCA2* were the most commonly mutated (7.9% of tumors), and were associated with increased TMB (Student’s t-test, p < 0.0001) (Supplementary Fig. S1a). Tumors with multiple DNA repair gene variants had corresponding increases in TMB. For example, tumors with 1 affected gene had an average of 293.8 ± 27.0 tumor mutations, while those with 3–5 affected genes had 815.8 ± 248.6 mutations (Fig. 1b). *POLE* variants were rare (n = 8) but were also associated with increased TMB (Student’s t-test, p = 0.010). There was no difference in smoking history between tumors with low and high TMB (Supplementary Fig. S1c).

We next calculated neoantigen burden in the samples by filtering total non-synonymous mutations based on predicted MHC binding affinity as a surrogate for immunogenicity. Strong putative binders were then further selected by assessing for poor immunogenicity of the non-mutated parental epitope. This total predicted neoantigen burden was significantly greater in tumors with somatic variants in HR (Student’s t-test, p = 0.0003) and MMR (p < 0.0001), but not POLE (p = 0.538) (Fig. 1c). Neoantigen burden was also positively associated with the number of affected DNA repair genes (Fig. 1d).

### High mutation burden is associated with increased tumor infiltration by activated T cells

**Figure 2:**
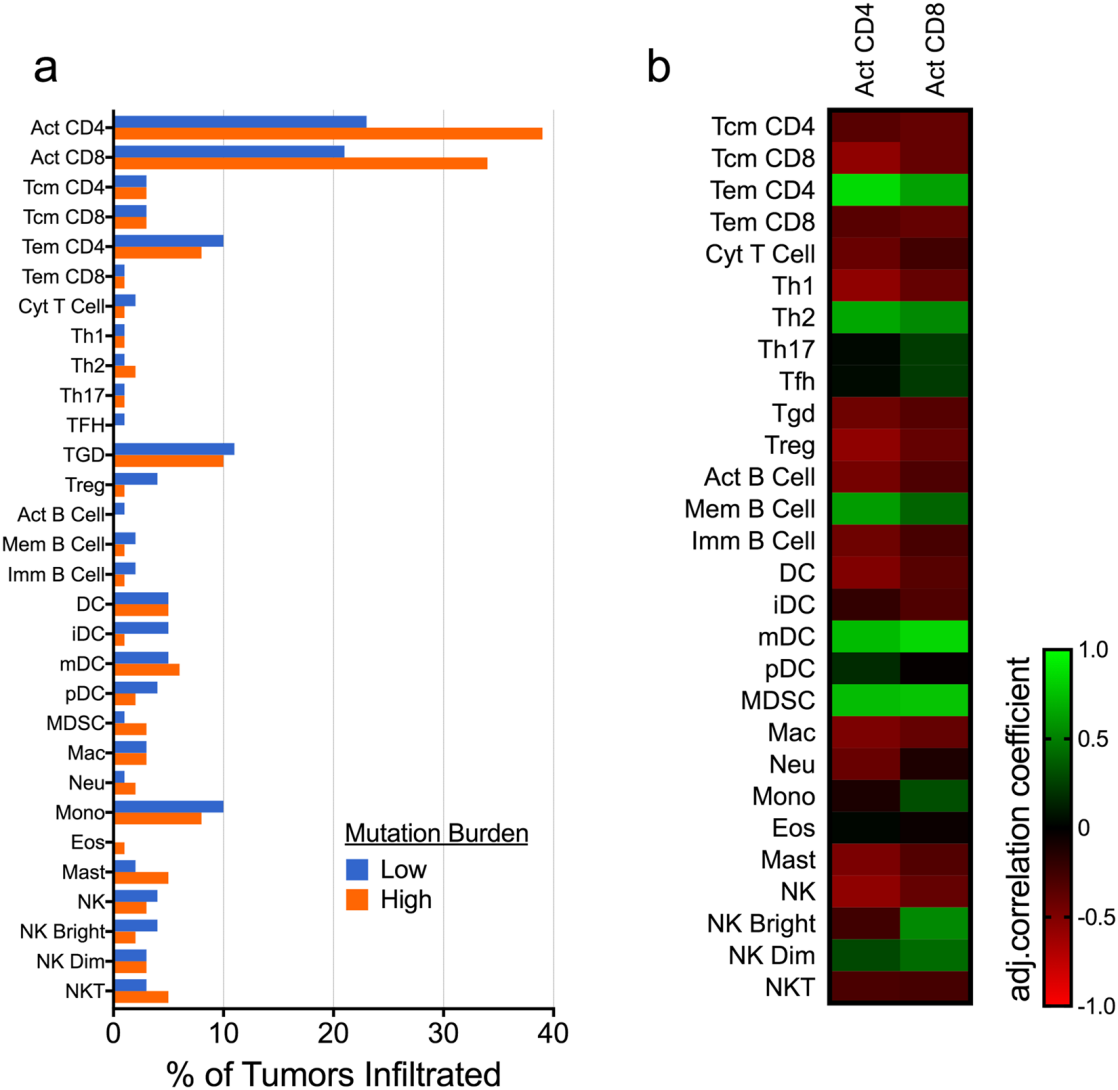
Activated T cell infiltration is increased in high-mutation tumors and associated with a specific immunophenotype. (**a**) Tumors with higher mutation burden were more likely to contain activated (Act) CD4+ and CD8+ activated T cells, though this difference was not significant after adjusting for multiple comparisons. (**b**) Activated CD4+ and CD8+ T cells were both positively correlated with CD4+ effector memory T cells (Tem), type 2 helper cells (Th2), memory (Mem) B cells, myeloid dendritic cells (mDC), and myeloid-derived suppressor cells (MDSC). Additional cell types analyzed include central memory T cells (Tcm); follicular helper T cells (Tfh); regulatory T cells (Tregs); gamma-delta T cells (Tgd); dendritic cells (DC); immature dendritic cells (iDC); plasmacytoid dendritic cells (pDC); macrophages (Mac); neutrophils (Neu); monocytes (Mono); eosinophils (Eos); mast cells (Mast); natural killer cells (NK); CD56 bright NK cells (NK Bright); CD56 dim NK cells (NK Dim); and natural killer T cells (NKT).

To determine the association between TMB and tumor-infiltrating lymphocytes (TILs), we divided TCGA samples into high- and low-mutation groups based on the median mutation count of 232. We then assessed immune cell infiltration from gene expression data as previously described^23^ (see Methods). Tumors with high TMB were more likely to be infiltrated by activated CD4+ (proportion Z-score, p = 0.013) and activated CD8+ (p = 0.036) T cells (Fig. 2a), but this finding was not statistically significant after adjusting for multiple comparisons (adjusted p = 0.38 and 0.52, respectively). Infiltration by activated CD4+ and CD8+ T cells was positively correlated with infiltration by CD4+ effector memory T cells, type 2 helper cells, memory B cells, myeloid dendritic cells, and myeloid-derived suppressor cells (Fig. 2b).

### DNA repair gene variants are not associated with T cell infiltration, possibly due to compensatory immunosuppressive signals

**Figure 3:**
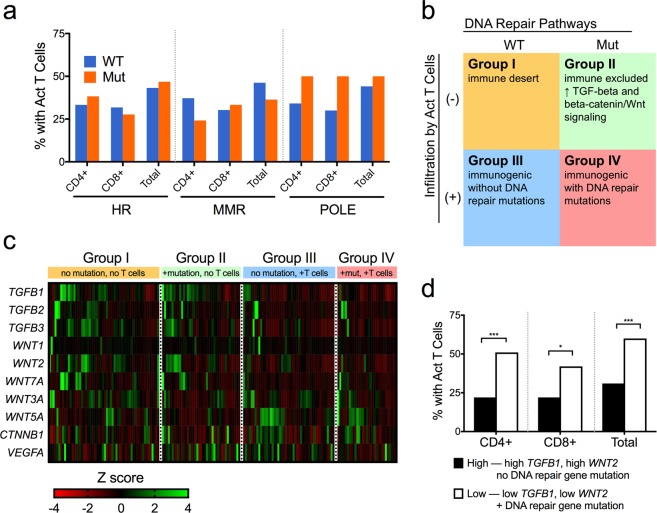
DNA repair gene variants and immune signals together predict infiltration by activated T cells. (**a**) DNA repair gene variants did not lead to a change in activated T cell infiltration. (**b**) These findings suggested that the tumors could be divided into four immunophenotypic groups based on DNA repair status and T cell infiltration. (**c**) A heat map of immunosuppressive genes showed differential mRNA expression among these groups. (**d**) A combined score incorporating DNA repair gene variants as well as *TGFB1* and *WNT2* expression was significantly associated with activated T cell infiltration (proportion Z-score, *FDR-adjusted p-value < 0.05, ***p < 0.001).

Presence of variants in HR, MMR, and *POLE* did not predict infiltration by CD4+, CD8+, or total activated T cells (Fig. 3a). To explain this finding, we categorized the samples into a 2 × 2 classification scheme based on DNA repair status and activated T cell infiltration (Fig. 3b). This grouping delineated four types of tumor: DNA repair variant absent without T cell infiltration (group I), DNA repair variant present without T cell infiltration (group II), DNA repair variant absent with infiltration (group III), and DNA repair variant present with infiltration (group IV).

We then evaluated the mRNA expression of genes previously shown to impair T cell infiltration, including those related to transforming growth factor beta (TGF-β) and the β-catenin/Wnt pathway^24^. Both group I and II tumors, which lack activated T cells, had significantly increased expression of TGF-β genes. Specifically, there were significant differences when comparing group II and IV tumors in regards to *TGFB1* (one-way ANOVA with Tukey’s test for multiple comparisons, p = 0.002) *TGFB3* (p = 0.049), and *WNT2* (p = 0.029) (Fig. 3c and Supplementary Fig. S2a). There was no significant association between groups based on *VEGF-A* expression (Fig. 3c and Supplementary Fig. S2a). We also performed a multivariate logistic regression analysis to determine the influence of these expression levels on group identity. When compared to group IV as a baseline, *TGFB1* and *WNT2* were significant predictors for groups I and II, while decreased levels of *WNT7A* was a significant predictor for group III (Table 1).

**Table 1:** Predictors of immunophenotypic group based on logistic regression analysis.

	Group I			Group II			Group III		
	Coef.	SE	p-value	Coef.	SE	p-value	Coef.	SE	p-value
*APC*	−0.166	0.235	0.480	0.104	0.228	0.649	−0.279	0.225	0.215
*CTNNB1*	−0.019	0.229	0.935	0.114	0.230	0.620	−0.015	0.217	0.944
*WNT1*	2.240	1.226	0.068	2.130	1.242	0.086	2.117	1.225	0.084
*WNT2*	0.970	0.410	0.018*	0.967	0.425	0.023*	0.331	0.437	0.449
*WNT3A*	0.141	0.235	0.547	−0.146	0.291	0.615	−0.032	0.237	0.891
*WNT5A*	0.232	0.277	0.401	−0.607	0.329	0.065	0.078	0.246	0.751
*WNT7A*	−0.323	0.303	0.286	−0.501	0.316	0.113	−0.828	0.402	0.039*
*TGFB1*	1.030	0.409	0.012*	1.341	0.421	0.001**	0.736	0.418	0.079
*TGFB2*	−0.232	0.179	0.195	−0.242	0.200	0.226	−0.318	0.219	0.145
*TGFB3*	0.097	0.355	0.785	−0.098	0.385	0.799	−0.034	0.390	0.930
*VEGFA*	0.203	0.234	0.385	0.321	0.235	0.172	0.312	0.216	0.148

A multinomial logistic regression model showed that *WNT2* and *TGFB1* had the strongest influence on determining group identity, using Group IV as a baseline. This table lists regression coefficients (Coef.) and standard errors (SE), *p < 0.05, **p < 0.01.

Presence of somatic variants in genes related to antigen presentation, including *HLA-A*, *HLA-B*, *HLA-C*, *B2M* (β-2-microglobulin), *TAP2*, and *PSMB8* (LMP-7), did not predict tumor classification (Supplementary Fig. S2b). Variants in the β-catenin/Wnt pathway were similarly not associated with tumor groups (Supplementary Fig. S2b).

### A combined score incorporating DNA repair status, *TGFB1*, and *WNT2* predicts T cell infiltration

As a proof of principle, we sought to compute a score based on the above data that could better predict T cell infiltration. Given the results of our logistic regression model, we conferred 1 point each for an expression z-score greater than the median for *TGFB1* and *WNT2*. We then added another point for absence of any DNA repair gene variants. Thus, a score of 3 indicates intact DNA repair pathways and high TGF-β/Wnt2, while a score of 0 indicates presence of DNA repair pathway variants and low TGF-β/Wnt2. We hypothesized that low scores would lead to increased T cell infiltration. Using a threshold of ≤1, tumors with low scores indeed demonstrated a significantly increased likelihood of CD4+ (false discovery rate-adjusted proportion Z-score, p < 0.0003) and CD8+ (p = 0.012) activated T cell infiltration (Fig. 3d).

We compared the expression of *TGFB1* and *WNT2* in tumors with and without activated T cells infiltration, after stratifying tumors based on DNA repair gene status (Supplementary Fig. S3a,b). *TGFB1* gene expression was significantly associated with activated CD4 and CD8 T cell infiltration regardless of DNA repair status, while *WNT2* was associated with T cell infiltration only in tumors with wild-type DNA repair genes (Supplementary Fig. S3a,b). Furthermore, we found that the combined score exhibited stronger correlations with immune cell infiltration than did either TMB or neoantigen burden (Supplementary Fig. S3c).

### Mutation and neoantigen burden are associated with increased expression of pro-inflammatory and immune checkpoint markers

**Figure 4:**
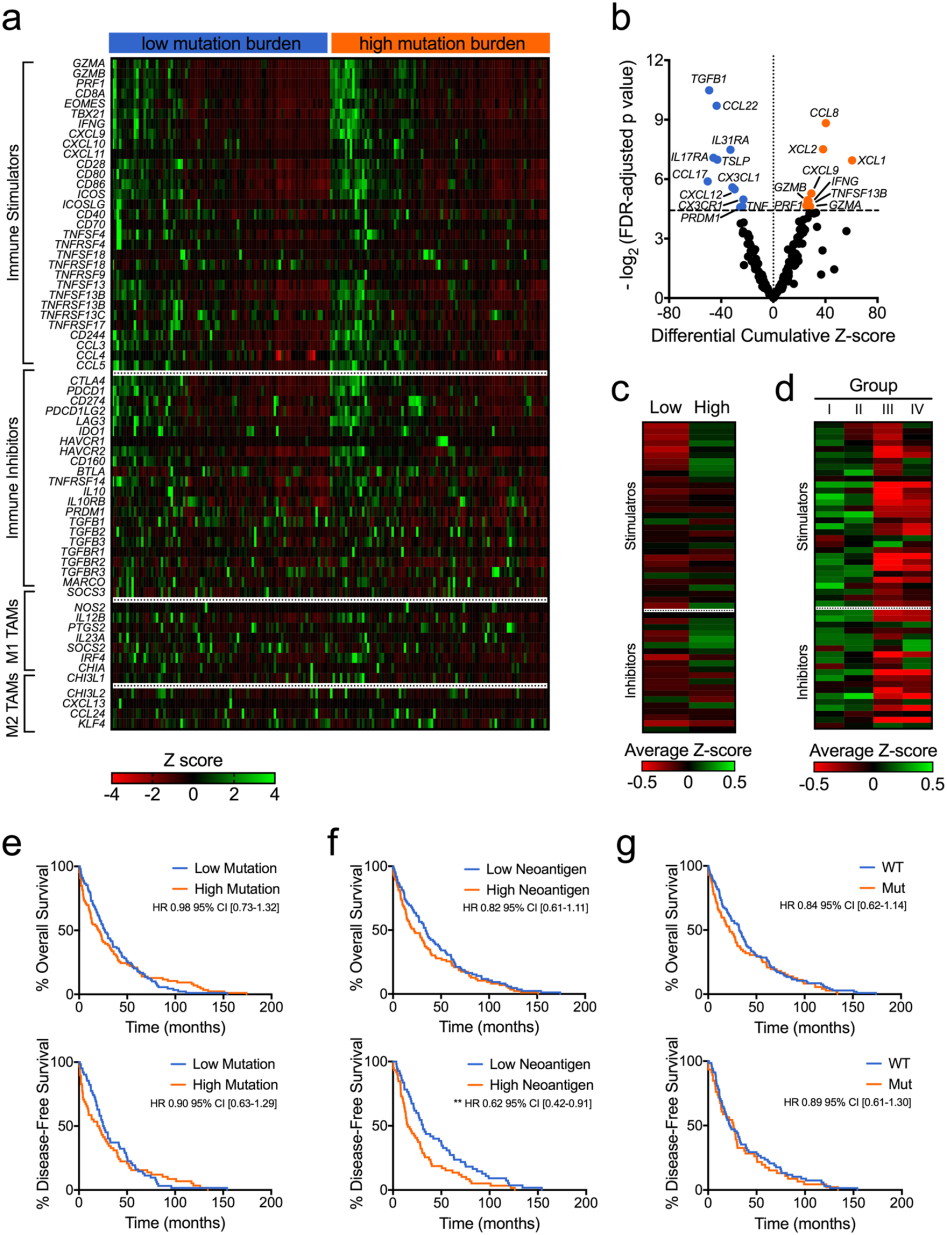
Mutation burden is associated with a unique immune and clinical profile. (**a**) High mutation burden was associated with an immune signature that includes increased expression of genes related to cytolytic and interferon-γ signaling. (**b**) A volcano plot shows differential mRNA expression in low versus high mutation tumors (Student’s t-test with FDR-adjusted p-values). Unique immune signatures were associated with (**c**) neoantigen load and (**d**) immunophenotypic group. (**e**) Mutation burden was not associated with significant differences in overall and disease-free survival. (**f**) High neoantigen burden was not associated with overall survival, but did lead to worse disease-free survival (**g**) Presence of DNA repair gene variants did not affect overall or disease-free survival. Kaplan-Meier plots shown, **p < 0.01 based on log-rank test.

We next utilized RNA-Seq expression data from TCGA to identify any potential immune signature related to increased tumor mutations and neoantigens. We first assessed associations between tumor mutation burden and mRNA levels of an immunomodulatory gene set^23^. SqCC samples with increased TMB had significantly increased expression of genes associated with immune activity, including *GZMA, GZMB, and PRF1* (Fig. 4a,b). They had higher levels of *IFNG* (interferon-γ) and the interferon-stimulated chemokine *CXCL9*. Meanwhile, high TMB tumors demonstrated decreased expression of *TGFB1* and *PRDM1* (Fig. 4a,b). Genes related to M1 and M2 tumor-associated macrophages (TAMs) did not have either higher or lower expression in these tumors (Fig. 4a).

In addition, we assessed for a comprehensive set of genes encoding immune-related cytokines and cytokine receptors. Expression levels of the cytokines *CCL8, CCL17, TNFSF13B, XCL1*, and *XCL2* were significantly increased in high TMB tumors (see Supplementary Fig. S4). Expression of the cytokines *CCL17*, *CCL22*, *CX3CL1, CXCL12, TNF*, and *TSLP*, as well as the cytokine receptors *CX3CR1*, *IL17RA*, and *IL31RA* were decreased in these tumors.

We subsequently classified SqCC samples by neoantigen load and immunophenotypic group and assessed the resulting immune signature. High-neoantigen tumors exhibited increased gene expression of the pro-inflammatory markers *GZMA*, *GZMB*, *PRF1*, *CD8A, EOMES*, *CXCL9*, and *IFNG*, as well as the immune checkpoint marker *LAG3* (Fig. 4c and Supplementary Table S1). The immunophenotypic groups (*i.e*. the classification based on DNA repair status and T cell infiltration) likewise demonstrated unique immune signatures (Fig. 4d and Supplementary Table S2). Notably, there was no significant difference in genes related to cytolytic activity, such as *GZMA*, *GZMB*, and *PRF1* (Supplementary Table S2). These groups did differ in expression levels of immune activation genes, including *CD28*, *CD80*, *CD86*, *ICOS*, *ICOSLG*, and *CD40*. They also had significant differences in regards to the immune checkpoint markers *CTLA4*, *PDCD1*, and *HAVCR2* (TIM-3).

Clinical analysis of this SqCC cohort has previously classified tumors into four subtypes that reflect their underlying biologic processes^25^. Mutation burden alone did not have strong association with any subtype (Supplementary Fig. S5a). However, MMR-variant tumors were more likely to have a secretory subtype (false discovery rate-adjusted proportion Z-score, p = 0.029) (Supplementary Fig. S5b).

### DNA repair status is not associated with overall survival

Mutation burden has been shown to affect both treatment response^26^ as well as intrinsic survival^27^ in multiple cancer types. In our cohort, overall survival was not affected by high mutation burden (HR 0.98 with 95% confidence interval 0.73–1.32) or neoantigen burden (HR 0.82 [0.61–1.11]) (Fig. 4e,f). Disease-free survival was not increased in the high TMB group (HR 0.90 [0.63–1.29]), but it was significantly improved in the high neoantigen group (HR 0.64 [0.44–0.93], p = 0.007). Presence of DNA repair variants was not associated with change in overall survival (HR 0.84 [0.62–1.14]) or disease-free survival (HR 0.89 [0.61–1.30]) (Fig. 4g). The immunophenotypic group also did not significantly affect survival outcomes (Supplementary Fig. S6a,b).

## Discussion

Defects in DNA repair pathways have been shown to strongly affect the tumor immune profile and consequently the clinical response to immunotherapy. We here sought to characterize the role of DNA repair gene mutations in shaping immunological characteristics in lung squamous cell carcinoma.

Our results overall support findings seen in other cancer types, and demonstrate that mutations in DNA repair pathways are associated with high tumor mutation burden (TMB). We also used mutation data to predict presence of tumor-specific neoantigens, which may be more relevant to anti-tumor immunity^28^, and showed that presence of DNA repair gene variants was associated with high neoantigen load. DNA repair status could thus serve as a surrogate marker for identifying patients with increased TMB. Recent clinical trials have demonstrated greater efficacy of immune checkpoint inhibitors in patients with NSCLC and high TMB^26,29^. Though targeted next-generation sequencing has been shown to be a viable method for measuring TMB^30^, assessing for mutation load remains a resource-intensive process. Use of more limited gene panels, such as one focused on DNA repair pathways, may be more practical for widespread clinical implementation.

An effective immune response requires not only the immunogenic impetus provided by tumor mutations, but also the ability of immune cells to infiltrate the tumor parenchyma^24,31^. In order to evaluate tumor infiltration, we utilized a comprehensive set of immune “metagenes” previously validated to estimate immune cell subpopulations^23^. In tumors with greater mutation load, there was a higher percentage of tumors infiltrated by activated CD4 + and CD8 + T cells. Though this difference was not statistically significant when adjusted for multiple comparisons, high TMB tumors had increased mRNA expression of genes that indicate T cell cytolytic activity, such as *GZMA* and *PRF1*^32^. High-mutation and high-neoantigen tumors also had significantly elevated expression of IFN-γ-inducible chemokines such as CXCL9, which promotes trafficking of activated T cells^33^. Furthermore, infiltration by activated T cells correlated with an increased presence of effector memory CD4 + T cells and myeloid dendritic cells, both of which play important roles in T cell stimulation and functionality^34,35^. These data together suggest that a real association between increased mutation load and T cell infiltration exists in lung SqCC.

Despite the relationship between mutation load and T cell infiltration, we did not find a direct association between DNA repair gene variants and tumor immunophenotype. Notably, there was a sizeable sub-group of tumors with repair gene variants (many with high mutation burden) that nonetheless did not exhibit T cell infiltration. This corresponds to prior categorizations of tumors into distinct categories based on the cancer-immunity cycle^24^: “immune desert” tumors with low neoantigen burden, “immune excluded” tumors that suppress T cell infiltration despite adequate neoantigens, and “inflamed” tumors with increased T cell activity. Our group of DNA repair gene-variant tumors without infiltrated T cells (*i.e*. “group II”) could thus represent an immune excluded phenotype.

Several factors have been postulated as contributing to immune exclusion. TGF-β represents a family of cytokines that regulate immune activity and have been demonstrated to reduce functionality of TILs^36,37^. High levels of TGF-β therefore represent a mechanism for tumors to impair immune cell infiltration despite the presence of adequate neoantigens. The β-catenin/Wnt pathway has also been implicated as contributing to decreased TILs and abrogating efficacy of immunotherapies^38^. In order to assess their potential role in lung SqCC, we looked at mRNA expression of TGF-β, β-catenin, and 5 Wnt genes known to be overexpressed in NSCLC^39^. We found that expression levels of genes related to TGF-β and the Wnt pathway were significantly increased in our putative immune excluded group. Deficiencies in the antigen presenting apparatus have been linked to low anti-tumor immune activity^40^, but mutations in relevant genes (including HLA molecules and β-2-microglobulin) were not predictive of immunophenotypic groups. HLA defects have been linked specifically to escape mechanisms in the context of acquired resistance to immunotherapy^41^, and they thus may not have relevance in a broader set of tumors.

By combining DNA repair status with expression of *TGFB1* and *WNT2* genes, we were able to calculate a multifactorial score that better predicted infiltration by activated T cells. This score is a crude measure but serves as a proof of concept demonstrating that DNA repair gene aberrations, when adjusted for tumor microenvironment factors, can help identify inflamed tumors. This finding does exist at odds with studies showing that MMR status alone can predict TIL presence and subsequent response to immunotherapies^15^. The use of somatic variants with unknown functional significance may have limited our analysis. However, it may also be that lung SqCC biology exhibits a greater propensity to create an immunosuppressive tumor milieu, especially when compared to colorectal^42^, endometrial^16^, and other cancer types whose immune responsiveness have been closely tied to DNA repair deficiencies.

In contrast to the results in SqCC, our prior study in lung adenocarcinoma did observe a direct relationship between DNA repair gene variants and activated T cell infiltration^19^. Important clinical differences have previously been observed between SqCC and adenocarcinoma; in particular, many recent advances in NSCLC treatment have had less impact on SqCC^1,43^. Nevertheless, these two subtypes frequently are grouped together, and few studies have explored the potential differences in their response to immune checkpoint inhibitors^6^. Our data imply that DNA repair status and TMB, when used as individual biomarkers, may be less predictive of immunophenotype in SqCC when compared to adenocarcinoma. Comprehensive immune signatures may instead be necessary to sufficiently capture relevant immune parameters in SqCC. In this dataset, we found significant differences between low- and high-mutation tumors in regards to immune-related gene expression, including in genes related to cytolytic activity and IFN-γ-inducible pro-inflammatory factors.

Increased mutation burden did not affect survival outcomes, but high neoantigen burden was associated with worse disease-free survival, possibly due to greater clinical relevance of tumor-specific neoantigens^7^. Meanwhile, presence of DNA repair gene variants did not relate to patient survival. This discrepancy seems to again imply that DNA repair status may have limited utility in SqCC. Variants in MMR genes, however, were associated with a secretory subtype, which interestingly has been defined by enhanced immune response^25^. It is possible that any clinical effect of DNA repair deficiencies would only be uncovered after treatment with immunotherapy. This SqCC cohort did not select for any treatment type, which may be relevant to several results. For example, we speculate that the link between neoantigen load and T cell infiltration might be stronger in a dataset obtained from immunotherapy responders. Similarly, high neoantigen load would be expected to lead to improved survival in those patients, while in these tumors, an increased number of neoantigens may represent more aggressive disease.

Identifying patients who will respond to immunotherapy, and tailoring treatments to their specific tumor biology, will rely on the ability to accurately assess the immune parameters of their tumors. To our knowledge, this work represents the largest analysis of the tumor immune profile in lung SqCC. Our data overall provide evidence that DNA repair pathway variants are closely associated with mutation and neoantigen burden in lung SqCC, and together with other immune-related signals are important factors in determining the tumor immunophenotype. Due to direct relevance of these parameters on the efficacy of immunotherapies, defects in DNA repair should be evaluated further as predictive biomarkers for these rapidly-developing treatments.

## Methods

### Data sets, mutation analysis, immune cell prediction

A previously annotated cohort of lung squamous cell carcinoma samples (n = 178) in The Cancer Genome Atlas (TCGA), as previously published^20^, was obtained from cBioPortal^44^. These data included DNA mutations, RNA-sequencing expression, and clinical descriptors. To identify mutations in homologous recombination, samples were assessed for mutations in the following genes: *ATR, ATM, CHEK1, CHEK2, BRCA1, BRCA2, BAP1, BARD1, FANCD2, FANCE, FANCC, FANCA, RAD50, RAD51*, and *PALB2*^45^. The mismatch repair pathway was assessed using the following gene list: *MLH1, MLH3, MSH2, MSH3, MSH4, MSH5, MSH6, PMS1, PMS2, PMS2L3, PCNA, EXO1, POLD1, RFC1, RFC2, RFC3, RFC4*, and *RFC5*^45^. All gene names are based on the HUGO Gene Nomenclature Committee (HGNC) database (https://www.genenames.org/)^46^. Gene transcripts were obtained using the HGNC-linked NCBI Reference Sequence (RefSeq) identifier^47^, with full data available at https://www.ncbi.nlm.nih.gov/refseq. Somatic variants in these genes were then filtered by including only those causing nonsense mutations, splicing errors, indels, or missense mutations predicted to be deleterious based on SIFT^21^ (sift.bii.a-star.edu.sg) score <0.05 or CADD v1.4^22^ (https://cadd.gs.washington.edu/) score >20. Splicing errors were defined as a 2-basepair variant in an intron adjacent to the intron/exon junction.

Infiltration of tumor samples by specific immune cell types was performed as previously described^23^. In brief, expression of 812 immune “metagenes,” which were derived from 813 microarrays over 36 studies, was entered into Gene Set Enrichment Analysis (GSEA)^48,49^ release 2.2.1. Any immune cell types with a false discovery rate (q-value) of ≤10% were considered as positively infiltrating into that tumor sample.

### Neoantigen prediction

Neoantigen prediction was done using CloudNeo pipeline (https://github.com/TheJacksonLaboratory/CloudNeo)^50^ on the NCI Cancer Genomics Cloud (CGC) in the Seven Bridges Genomics implementation. A list of non-synonymous mutations in maf file format was downloaded from TCGA which was converted into the vcf file format (https://github.com/mskcc/vcf2maf/blob/master/maf2vcf.pl). The genomic variants were translated into amino acid changes using the Variant_Effect_Predictor (release-83) tool^51^ and a custom script in the CloudNeo pipeline^50^ using the programming language R (version 3.5.1, https://www.r-project.org). The output of the custom tool is a list of N-amino-acid-long peptide sequences in a fasta format, such that the single peptide change is in the middle of the N-mer. The HLA prediction was done using the HLAminer tool version v1.3^52^, which takes HLA allele database file which can be downloaded from the CloudNeo github (http:/cloudneo.readthedocs.io/en/latest/cloudneo/inputs.html).

Peptides of 9 amino acids containing mutated sites were tested against 6 predicted HLA types using the CloudNeo pipeline in order to generate a neoantigen affinity score using the Netmhcpan (v3.0a) tool^53^. A control analysis was performed with the homologous non-mutated peptides. Neoantigens were identified as mutated peptides with strong binding affinity, as defined by IC50 <500 nM, with positive gene expression and corresponding non-mutated wild-type peptides with weak MHC binding (IC50 > 500 nM). Supplementary Fig. S7 displays the representative schematic diagram of the CloudNeo pipeline along with the actual commands that were invoked for a sample to illustrate the parameter settings for various tools within the pipeline. Please note that we have substituted our actual project and sample path with simple strings.

### Statistical analysis

Comparison of data was carried out using the z-score between two population proportions, unpaired two-tailed Student’s or Welch’s *t*-test, ANOVA analysis, and the Pearson correlation coefficient as appropriate. These calculations were performed in GraphPad Prism version 7 (La Jolla, CA). P-values obtained by ANOVA were adjusted for multiple comparisons using Tukey’s test. Adjustment for false discovery rate (FDR) was performed using Storey’s q-value estimation in R^54^. Logistic regression analysis was also performed using R. Results are presented as percentages, means ± standard error of the mean (SEM), Pearson correlation coefficient, or RNA-Seq z-score, as indicated. P-values are represented by *p < 0.05, **p < 0.01, ***p < 0.001, and ****p < 0.0001.

## Supplementary information

Supplementary Figures

Dataset 1

## Data Availability

The datasets analyzed during the current study are available in the cBioPortal repository of The Cancer Genome Atlas, found at http://www.cbioportal.org, last accessed 10/28/2018. Lists of primary variants for tumors are available from The Cancer Genome Atlas at the National Institutes of Health website, found at https://tcga-data.nci.nih.gov/docs/publications/lusc_2012/, last accessed 10/26/2018.
