# Supplementary material for: Mutations in DNA repair genes are associated with increased neoantigen burden and a distinct immunophenotype in lung squamous cell carcinoma
Source: Sci Rep. 2019 Mar 1;9:3235. doi: 10.1038/s41598-019-39594-4 (PMC6397194; doi:10.1038/s41598-019-39594-4)
Supplement: Supplementary file 1 — Supplementary Figures [file 41598_2019_39594_MOESM1_ESM.pdf]

Supplementary Figures and Tables

Mutations in DNA repair genes are associated with increased neoantigen burden and a distinct immunophenotype in lung squamous cell carcinoma

Young Kwang Chae[1,2]*, Jonathan F. Anker[1]*, Michael S. Oh[1]*, Preeti Bais[3], Sandeep Namburi[3], Sarita Agte[1], Francis J. Giles[1,2], Jeffrey H. Chuang[3,4]

Supplementary Figure 1
(a) Mutations in *BRCA1* and *BRCA2* were associated with increased mutation count as well as (b) increased neoantigen count. Statistical analysis completed with Student's t-test, *** $p < 0.001$, **** $p < 0.0001$. (c) Mutation burden was not significantly associated with patient smoking history.

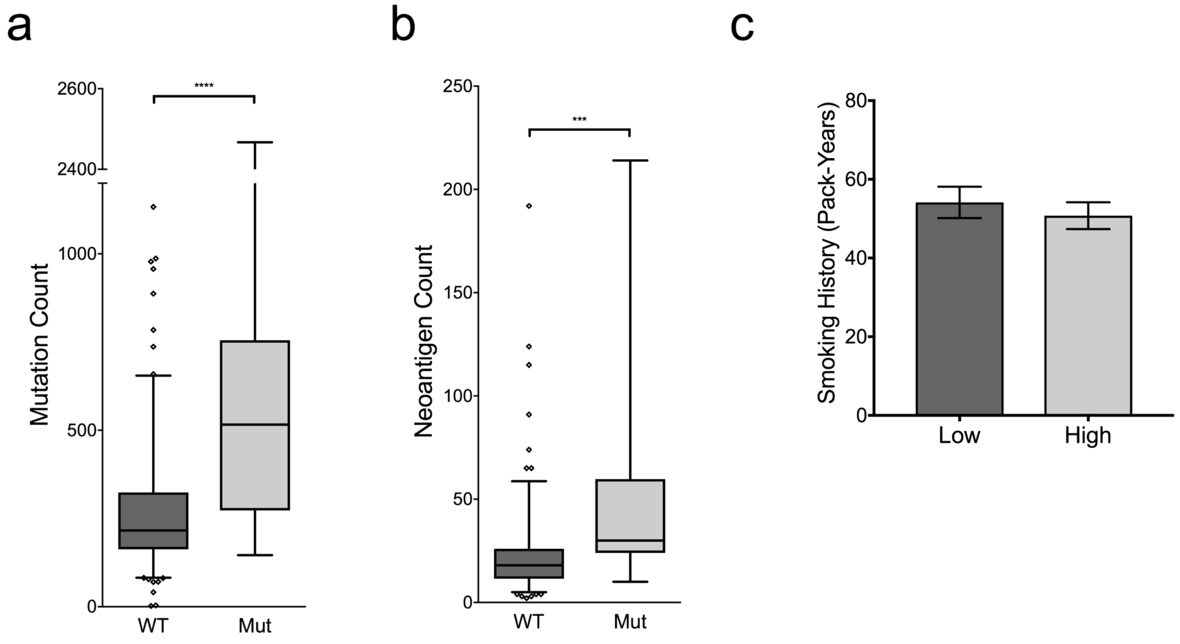

Supplementary Figure 2

(a) *TGFB1*, *TGFB3*, *WNT2*, and *WNT5A* had significant differences in expression between immunophenotypic groups (one-way ANOVA with Tukey's test for multiple comparison, * $p < 0.05$, ** $p < 0.01$, *** $p < 0.001$). (b) Mutations in Wnt and antigen presentation genes are depicted based on tumor groups. Samples within each group are arranged from highest mutation burden on the left to lowest mutation burden on the right.

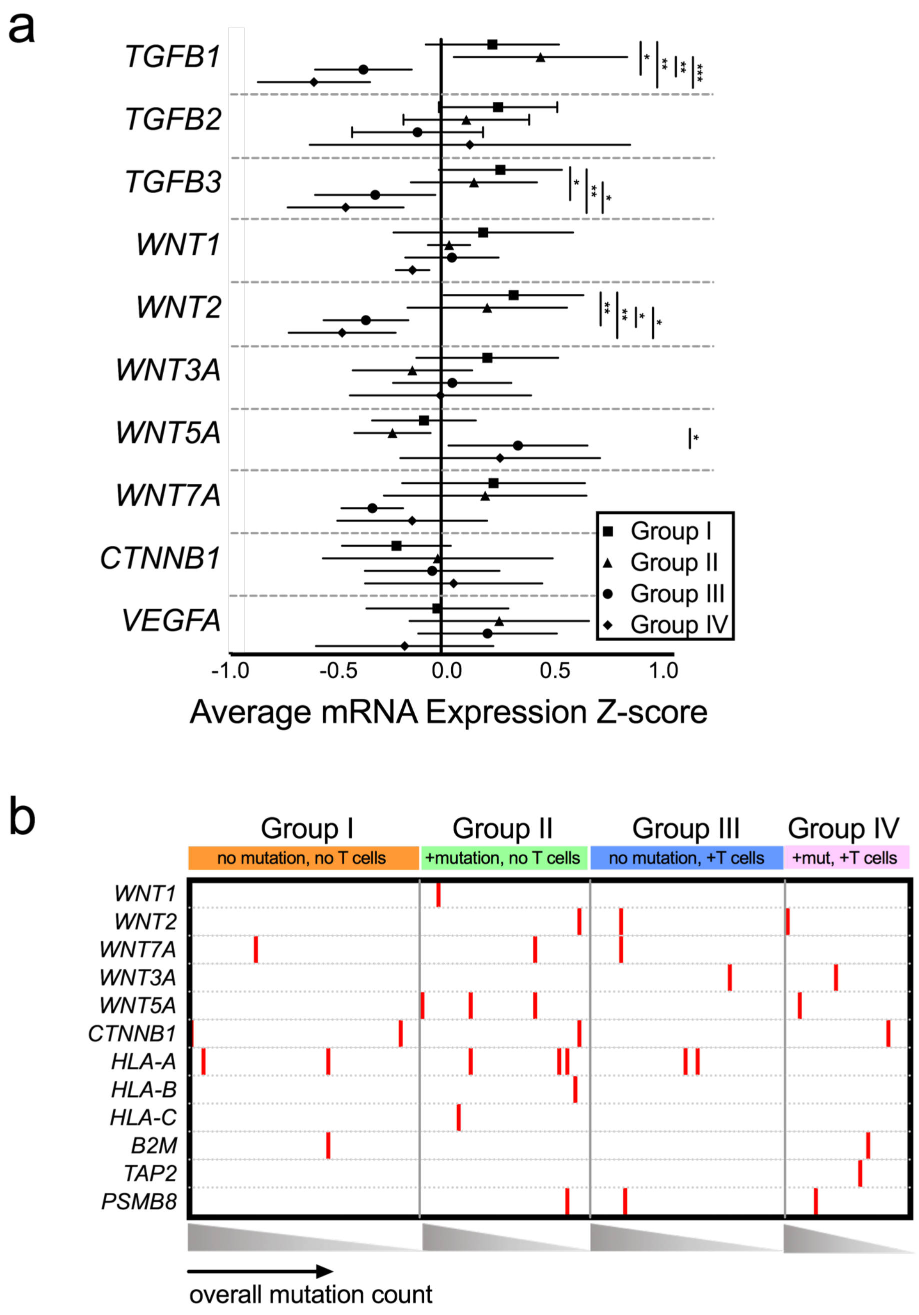

Supplementary Figure 3

(a) Two-dimensional plots show tumors color-coded based on activated CD4 and (b) CD8 infiltration. Samples are plotted based on *TGFB1* and *WNT2* expression, and stratified based on presence of DNA repair gene variants. P-values refer to gene expression differences in tumors with and without T cell infiltration (using Welch's two-sample t-test). (c) A heat map shows Pearson correlation coefficients between immune cell infiltration and either combined score, neoantigen burden, mutation burden, or repair gene mutations.

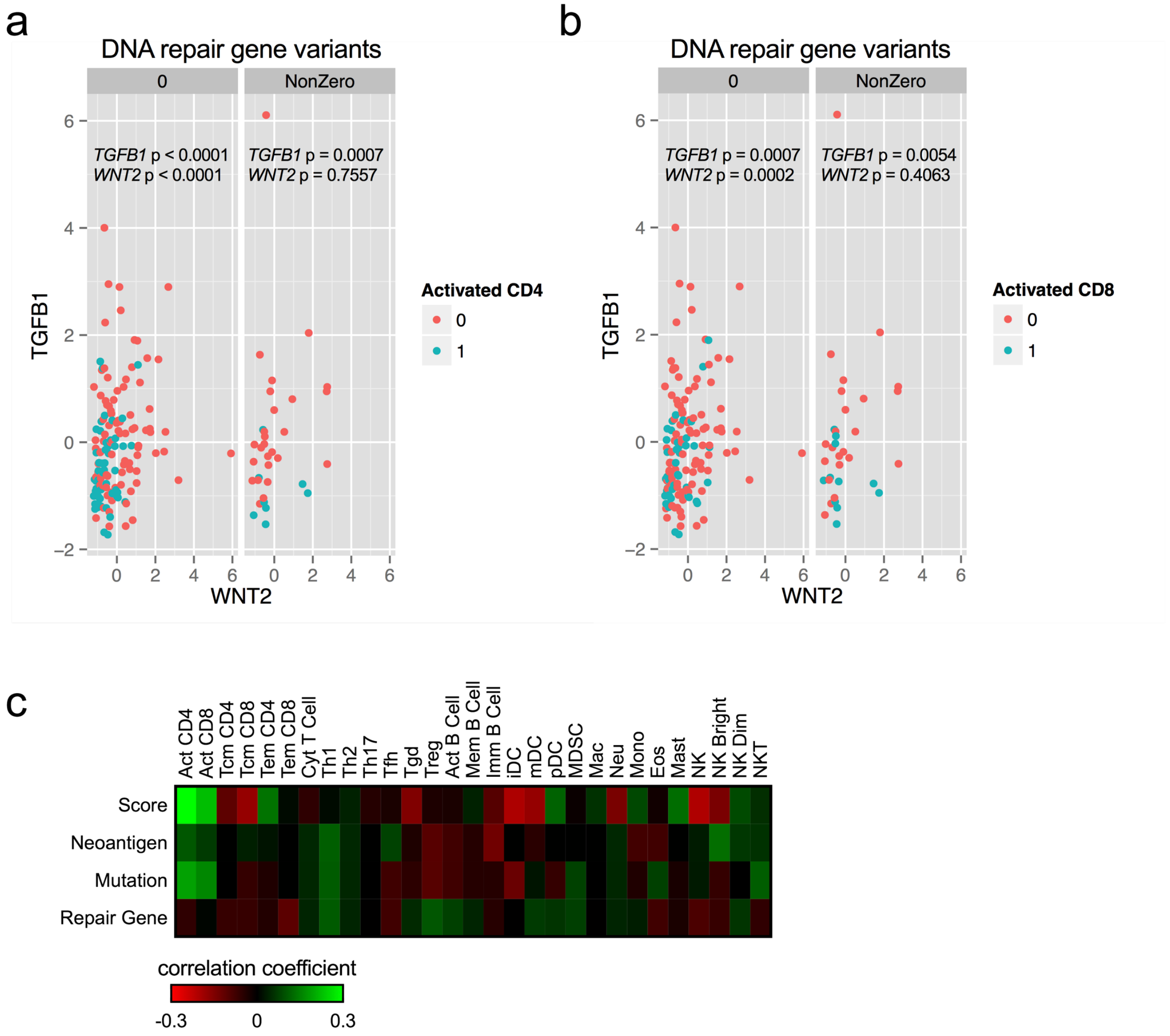

Supplementary Figure 4

Heat map of mRNA expression of additional immune-related genes. Genes are ordered based on difference in average expression between high and low mutation burden tumors.

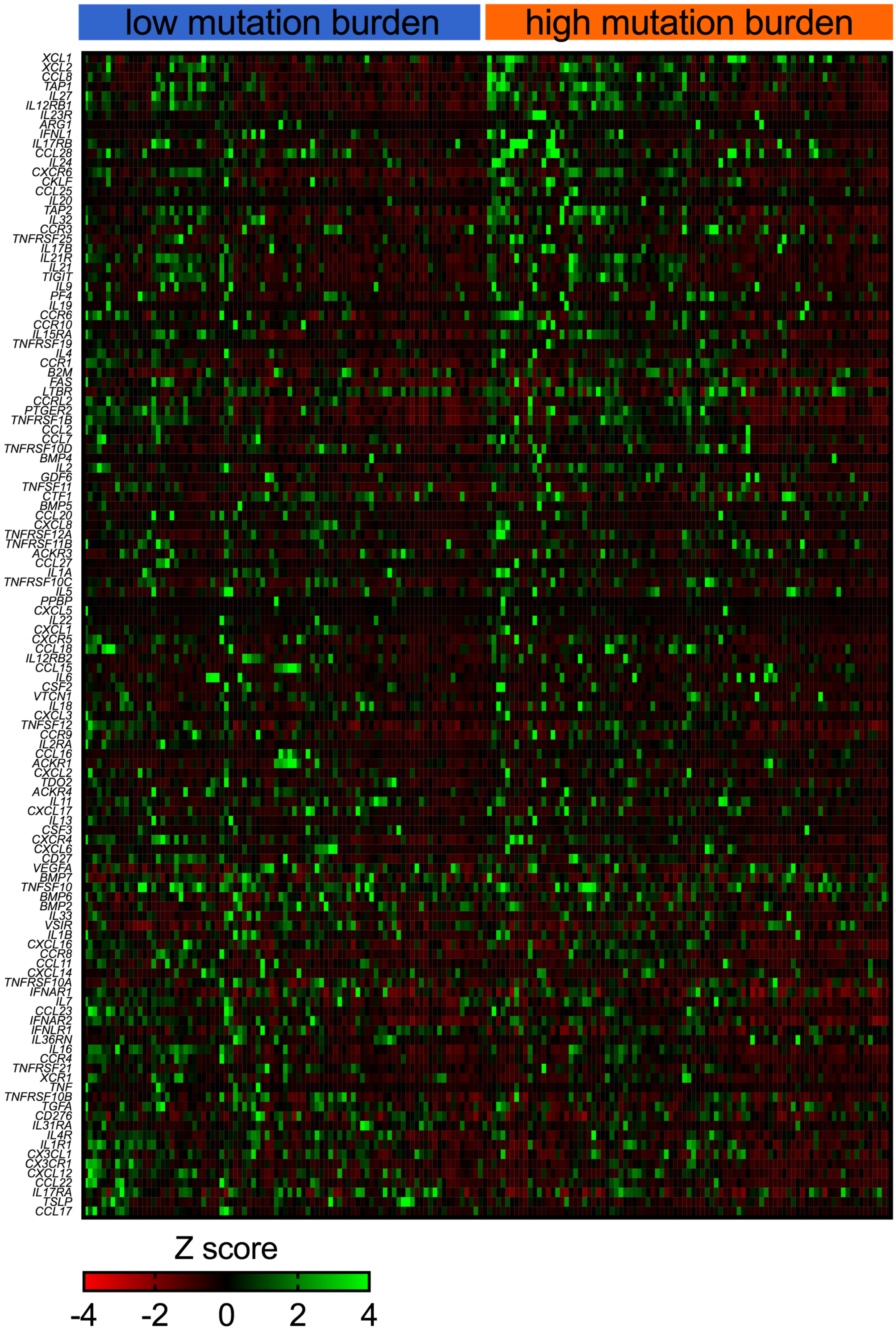

Supplementary Figure 5
(a) Mutation burden was not associated with histological subtypes of squamous cell carcinoma. (d) Variants in MMR genes were associated with greater proportion of tumors with secretory subtype (proportion Z-score, * FDR-adjusted p < 0.05).

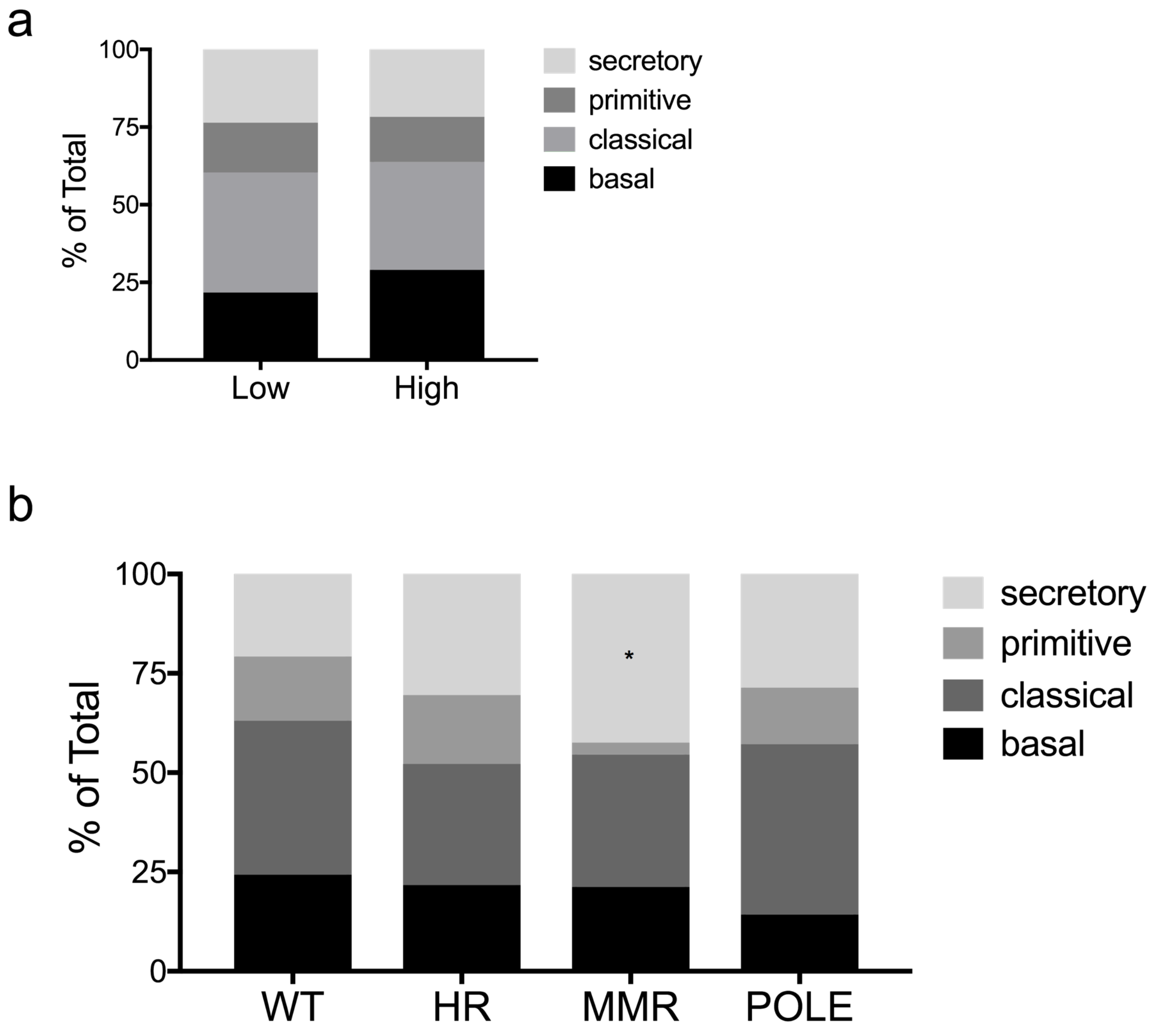

Supplementary Figure 6

Kaplan-Meier plots demonstrating (a) overall survival (OS) and (b) disease-free survival (DFS) in patients divided based on tumor groups (as defined in Figure 3b). There was no statistically-significant difference between groups based on log-rank test with either OS or DFS.

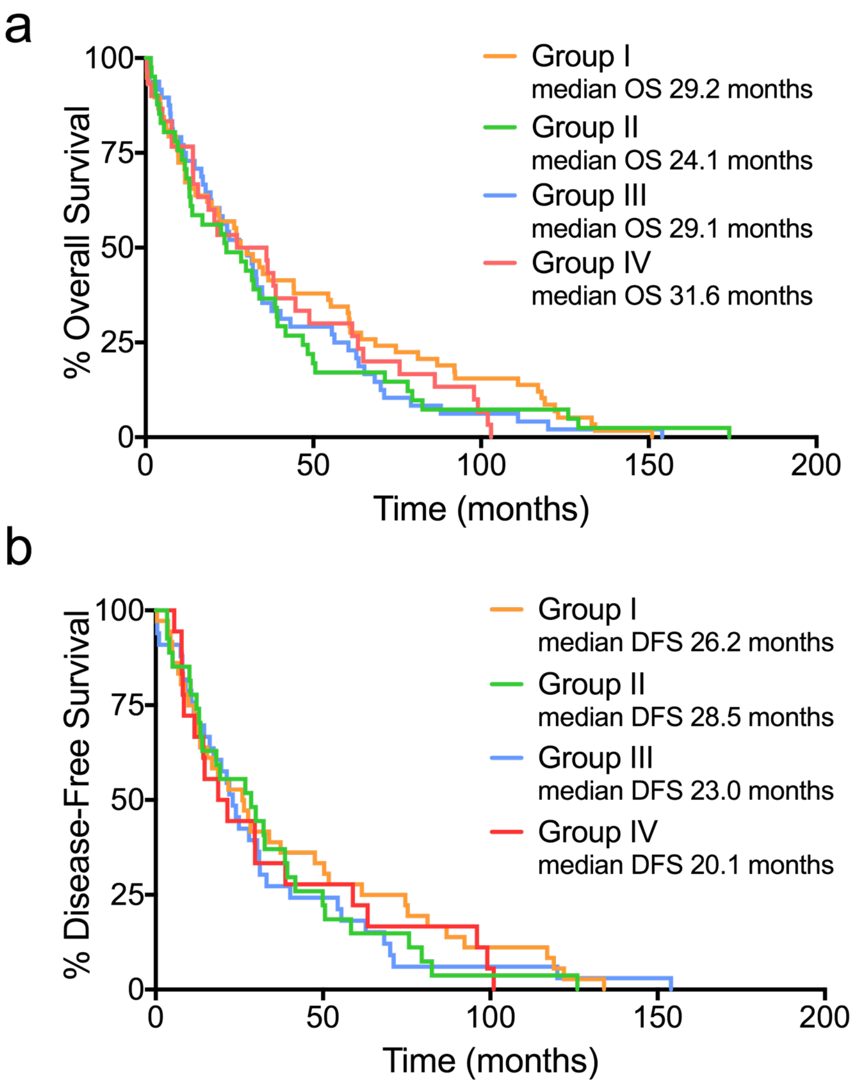

Supplementary Figure 7

Representative schematic diagram of the CloudNeo pipeline, including the commands that were invoked to illustrate the parameter settings for various tools within the pipeline. Please note that we have substituted our actual project and sample path with simple strings. In addition, please note that the “Specification of Peptides” step generates both the tumor and control peptide files in fasta format and the “Neoantigen Prediction” step is run for both of them separately.

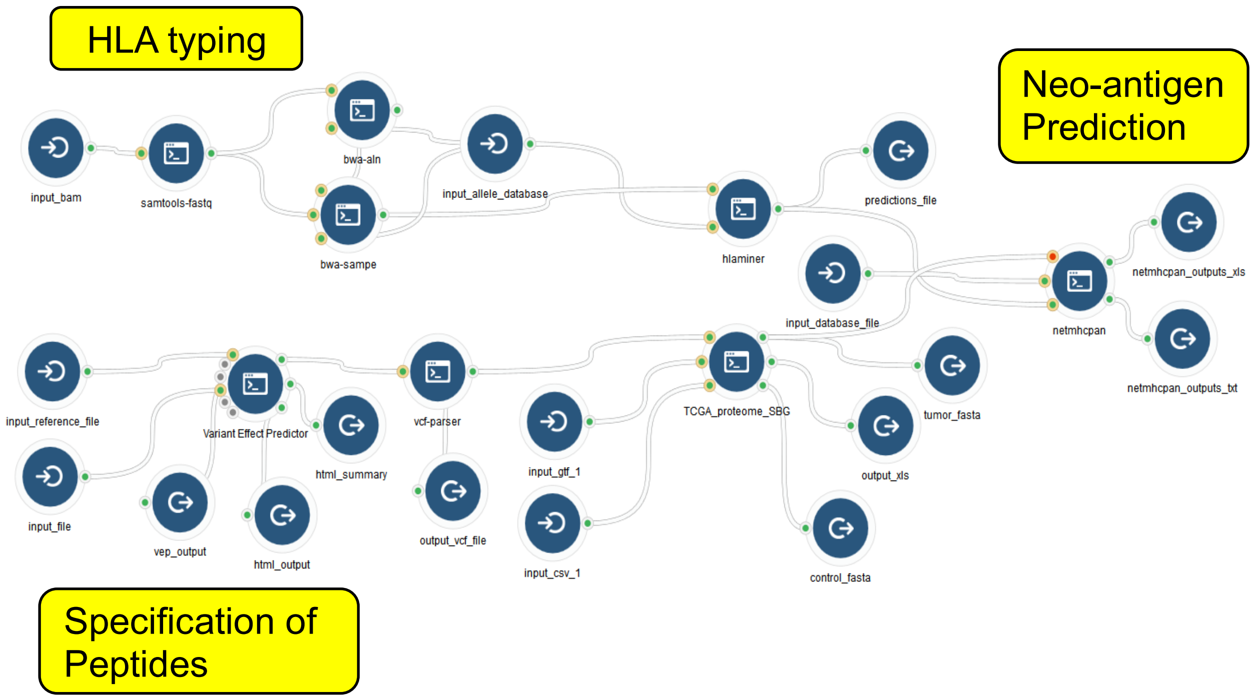

Supplementary Table 1. Significance values for comparison of immune signature in low versus high neoantigen tumors. Statistical analysis performed using Student's t-test with Storey's adjustment for false discovery rate (FDR) with multiple comparisons (* $p < 0.1$)

	FDR-adj. p
GZMA	0.0575*
GZMB	0.0633*
PRF1	0.0575*
CD8A	0.0575*
EOMES	0.0675*
TBX21	0.1128
IFNG	0.0575*
CXCL9	0.0633*
CXCL10	0.5298
CXCL11	0.2274
CD28	0.6327
CD80	0.6030
CD86	0.6030
ICOS	0.6327
ICOSLG	0.6418
CD40	0.6357
CD70	0.2334
TNFSF4	0.6030
TNFRSF4	0.6030
TNFSF18	0.6327
TNFRSF18	0.6327
TNFRSF9	0.6030
TNFSF13	0.6327
TNFSF13B	0.4342
TNFRSF13B	0.6327
TNFRSF13C	0.6327
TNFRSF17	0.6327
CD244	0.3879
CCL3	0.6327
CCL4	0.6030
CCL5	0.0575*
CTLA4	0.6327
PDCD1	0.1291
CD274	0.6327
PDCD1LG2	0.1668
LAG3	0.0575*
IDO1	0.6327
HAVCR1	0.6030
HAVCR2	0.2546
CD160	0.1668
BTLA	0.3636
TNFRSF14	0.6327
IL10	0.2546
IL10RB	0.6327
PRDM1	0.6327
TGFB1	0.1790
TGFB2	0.3901
TGFB3	0.6357
TGFBR1	0.4342
TGFBR2	0.6030
TGFBR3	0.6030

Supplementary Table 2. Significance values for comparison of immune signature between immunophenotypic groups. Statistical analysis performed using one-way ANOVA with Storey's adjustment for false discovery rate (FDR) with multiple comparisons (* p < 0.05, ** p < 0.01, *** p < 0.001, **** p < 0.0001).

	F	*p-value*	*FDR-adj. p*
GZMA	1.606	0.1899	0.1621
GZMB	1.973	0.1198	0.1159
PRF1	2.484	0.0625	0.0725
CD8A	1.518	0.2115	0.1735
EOMES	2.507	0.0606	0.0725
TBX21	5.136	0.0020	0.0036**
IFNG	0.6953	0.5561	0.3842
CXCL9	1.691	0.1706	0.1500
CXCL10	1.504	0.2152	0.1735
CXCL11	0.01778	0.9968	0.5672
CD28	13.86	<0.0001	<0.0001****
CD80	5.622	0.0011	0.0025***
CD86	13.3	0.0001	0.0003***
ICOS	7.642	<0.0001	0.0003***
ICOSLG	3.735	0.0123	0.0188*
CD40	5.34	0.0015	0.0031**
CD70	0.9753	0.4057	0.2872
TNFSF4	3.963	0.0092	0.0148*
TNFRSF4	3.263	0.0228	0.0315*
TNFSF18	1.16	0.3267	0.2385
TNFRSF18	2.075	0.1052	0.1053
TNFRSF9	1.889	0.1333	0.1209
TNFSF13	12.79	<0.0001	<0.0001****
TNFSF13B	7.266	0.0001	0.0003***
TNFRSF13B	8.911	<0.0001	0.0001***
TNFRSF13C	1.155	0.3287	0.2385
TNFRSF17	5.302	0.0016	0.0031**
CD244	2.615	0.0527	0.0665
CCL3	1.911	0.1296	0.1209
CCL4	0.5231	0.6672	0.4303
CCL5	2.88	0.0374	0.0493*
CTLA4	5.755	0.0009	0.0024**
PDCD1	5.01	0.0023	0.0039**
CD274	0.1594	0.9235	0.5360
PDCD1LG2	0.644	0.5877	0.3876
LAG3	2.109	0.1008	0.1045
IDO1	0.1615	0.9221	0.5360
HAVCR1	1.442	0.2322	0.1821
HAVCR2	9.606	<0.0001	0.0001***
CD160	2.248	0.0845	0.0943
BTLA	1.262	0.2891	0.2208
TNFRSF14	9.437	<0.0001	0.0001***
IL10	3.647	0.0138	0.0200*
IL10RB	0.1997	0.8965	0.5360
PRDM1	2.147	0.0961	0.1033
TGFB1	8.34	<0.0001	0.0001***
TGFB2	0.4481	0.7189	0.4535
TGFB3	5.649	0.0010	0.0024**
TGFBR1	0.1696	0.9168	0.5360
TGFBR2	8.242	<0.0001	0.0002***
TGFBR3	0.6525	0.5824	0.3876
